# Diversification dynamics of total-, stem-, and crown-groups are compatible with molecular clock estimates of divergence times

**DOI:** 10.1126/sciadv.abf2257

**Published:** 2021-06-11

**Authors:** Alan J. S. Beavan, Davide Pisani, Philip C. J. Donoghue

**Affiliations:** 1 School of Biological Sciences, University of Bristol, Life Sciences Building, Tyndall Avenue, Bristol BS8 1TQ, UK.; 2 School of Earth Sciences, University of Bristol, Life Sciences Building, Tyndall Avenue, Bristol BS8 1TQ, UK.

## Abstract

Molecular evolutionary time scales are expected to predate the fossil evidence, but, particularly for major evolutionary radiations, they can imply extremely protracted stem lineages predating the origin of living clades, leading to claims of systematic overestimation of divergence times. We use macroevolutionary birth-death models to describe the range of total-group and crown-group ages expected under constant rates of speciation and extinction. We extend current predictions on origination times for crown- and total-groups, and extinction of stem-groups, demonstrating that there is broad variance in these predictions. Under constant rates of speciation and extinction, we show that the distribution of expected arthropod total-group ages is consistent with molecular clock estimates. The fossil record cannot be read literally, and our results preclude attempts to interpret the antiquity of clades based on the co-occurrence of stem- and crown-representatives.

## Teaser

A wide range of divergence times is supported by birth-death models under constant rates of speciation and extinction.

## INTRODUCTION

One of the principal goals of paleontology has been to establish a time scale for the tree of life (*1*), facilitating estimates of evolutionary rates and temporal tests of evolutionary causality. The earliest fossil representative of a clade clearly demonstrates its establishment but may not approximate clade age very closely. Molecular clock methodology attempts to estimate clade age directly by combining fossil constraints on clade age with a phylogenetic hypothesis, molecular sequence data, and a model of molecular evolution. Early applications of the molecular clock, which assumed a constant evolutionary rate, invariably estimated clades to be markedly older than the oldest fossil evidence, particularly for major evolutionary radiations, such as the diversifications of animals, modern mammals, birds, and flowering plants. The introduction of evolutionary models incorporating rate variation across lineages and the uncertainty associated with fossil calibration (*2*–*5*) has diminished the disparity between molecular clock estimates and the fossil record, but for these iconic evolutionary radiations, a significant gap remains. Some suggest that this might be because early representatives of a clade are low in abundance, ecologically restricted, unlikely to fossilize, or are lost to the rock record [see, e.g., (*6*, *7*)], while others claim that molecular divergence times are gross overestimates. Thus, for example, the diversification of bilaterian animals is alternately perceived to have been a Cambrian explosion based on an approximately literal reading of the fossil record versus a more protracted Precambrian diversification based on molecular divergence times (*7*).

Reconciling these competing perspectives is challenging because the true time scale is never known. However, forward modeling of macroevolutionary dynamics can be used to explore the probability of different hypotheses. The benefit of modeling is that analyses can be repeated thousands of times, generating a sample large enough to make confident interpretations concerning the statistical properties of the results. Furthermore, the parameters of the model can be modified to explore their effect. If a divergence time is very unlikely under an evolutionary model, then an alternative model is needed to explain it; otherwise, the model can be considered satisfactory to explain empirical data.

Budd and Mann (*8*) implemented a birth-death model using constant rates of speciation (birth) and extinction (death) to characterize the dynamics of clades. The evolutionary history of a living clade can be divided into different episodes with respect to the nearest living relatives: The crown-group is composed of its living members, their common ancestor, and all its extinct descendants; the stem-group is composed of extinct lineages more closely related to the crown-group than any other living clade; and the total-group is the sum of the stem- and crown-groups. Budd and Mann (*8*) infer that, in the absence of mass extinction events or changes in speciation and extinction rates, presumptive crown-groups originate early in the evolution of their total-group, after which lineages in the presumptive stem-group are quickly extinguished. They interpret the fossil record of flowering plants, birds, and especially bilaterians in this light: The co-occurrence of stem- and crown-representatives in the early fossil record of these clades precludes anything more than a very short prehistory of the total-group (*8*). This, they suggest, is evidence that the time scale of animal evolution is approximated much more closely by the fossil record than molecular clock estimates imply. Budd and Mann (*8*) did not use a simulation approach to derive these interpretations. Instead, they mathematically derive the probabilities of macroevolutionary phenomena, such as the age of the crown-group and stem-group diversity through time, often using fixed values for crown-group and total-group ages. Here, we use a mixture of simulations and mathematical derivations similar to those used by Budd and Mann (*8*) to relax these fixed values and explore more fully the distributions of crown-group and total-group ages that can be generated under a model of constant speciation and extinction. We also consider the full distribution of stem-groups to explore statistically the timing of stem-group extinction relative to crown-group origination. The results of our study reveal a much broader distribution of age relationships between the crown- and total-groups and between the origin of crown-groups and the extinction of their respective stem-groups. On this basis, we reject Budd and Mann’s approach of inferring the timing of origin of major clades based on the temporal range of their stem- and crown-groups not because their results are wrong, but because they do not explore the full range of divergence times that are feasible. Using constraints on the diversity and antiquity of crown-arthropods, we find a distribution of age estimates for the total-group in agreement with molecular clock estimates, highlighting the importance of investigating variance around estimates in avoiding making overconfident interpretations.

## RESULTS

### The emergence of the crown-group is variable

**Fig. 1:**
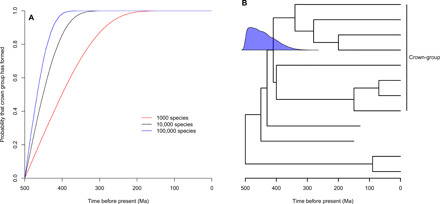
Times of crown-group origin under a fixed total-group age. (**A**) Cumulative probability of the crown-group origin is plotted against time on the *x* axis in simulated total-groups with 1000, 10,000, and 100,000 species originating at 500 Ma. (**B**) A hypothetical tree showing the distribution of crown-group ages when 10,000 tips are generated in a total-group originating at 500 Ma. All taxa in the crown-group are labeled.

First, we asked how the ages of crown-groups are distributed under a fixed total-group age, with constant rates of speciation and extinction, generating varying numbers of species. This addresses the timing of origin of crown-groups relative to their total-groups. Initially, trees with no fixed topology, generating 10,000 species, were simulated using an extinction rate of 0.5 extinctions per species per million years, a total-group origin of 500 million years before present (Ma), and a speciation rate of 0.5107 events per species/million years, which is the maximum likelihood inferred speciation rate, given the other parameters. The mean crown-group age was found to be 444.7 Ma; the median is 451.1 Ma; and in 95% of cases, the crown-group emerged within the interval 497.7 to 357.1 Ma [sample size (*n*) = 37,000; Fig. 1].

Simulations were also performed using the same time parameters but different numbers of tips and speciation rates. When trees with 1000 tips were evolved within the same time period, the crown emerged between 495.1 and 233.6 Ma in 95% of simulations (speciation rate = 0.505, *n* = 291,191). When 100,000 tip trees were simulated, 95% of the crown-groups originate within 498.4 to 403.6 Ma (speciation rate = 0.516, *n* = 4,907; Fig. 1).

We investigated how the distribution of crown-group ages would change if evolution was given another 500 million years (i.e., for a total of 1 billion years). This experiment informs on whether the age of the crown-group varies with lineage longevity. In simulations with trees of 100,000 tips, extending the duration of the evolutionary process (while keeping the other parameters constant) did not increase the absolute duration of the stem lineage (age of the total-group minus that of the crown-group). In simulations with 10,000 tip trees, the crown-group age changed in 0.88% of simulations. Of these simulations, 95% of the stems changed by less than 99.7 million years. In the simulations with 1000 tip trees, there were a very small number of runs (0.001%) where the clade went extinct. In the remaining simulations, the crown-group age changed in 19.5% of the considered cases, among which 95% of stem lengths changed by up to 228.8 million years.

### Stem-group extinction varies stochastically and depends on the crown-group origin

Next, we investigated when stem-groups go extinct under constant rates of speciation and extinction. To do this, we draw information about the extinction of stem-groups from mathematically derived probability distributions outlined by Budd and Mann (*8*). Here, crown-groups were forced to emerge within the range of ages observed in our first set of simulations. In all cases, the trees had 10,000 tips, the extinction rate was 0.5 events per species/million years, and the speciation rate was 0.5107 events per species/million years. The total-group was fixed at 500 Ma. Crown origins were fixed at 497.7 and 357.1 Ma, representing the upper and lower 95% bounds in our previous crown-group age simulations. In addition, we included analyses with the crown-group originating at 410 Ma, which was the value used by Budd and Mann (*8*), as well as at multiples of 20 million years for 120 million years after the origin of the total-group (480 to 380 million years).

**Fig. 2:**
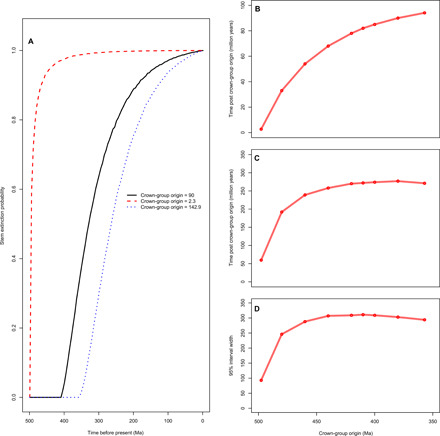
Probabilities of stem-group extinction through time. (**A**) The probability of the stem being extinct on the *y* axis is shown against time on the *x* axis. The crown-groups are fixed at times given by the key in the bottom right of the pane. (**B**) The time that has passed since crown-group emergence at which the probability that the stem-group is extinct is 50% against time of crown-group origin on the *x* axis. (**C**) The time after the crown-group formation at which the stem-group is 95% likely to be extinct against time of crown-group origin. (**D**) The size of the range of time after the crown-group forms between which the stem-group is 95% likely to go extinct.

For each crown-group origin, we followed the pipeline of Budd and Mann (*8*). We drew numbers of stem lineages at 500 time points from 0 to 500 Ma from mathematically derived probability distributions and calculated the probability that the stem-group had gone extinct at each time point according to 50,000 repeated analyses. The cumulative probability of the stem-group going extinct when the crown-group was fixed at 497.7, 410.0, and 357.1 Ma (Fig. 2A) shows that as the time after the crown-group origin increases, so does the likelihood of stem-group extinction, necessarily reaching 1 by the end of the simulation. The time after the origin of the crown-group at which the probability of the stem-group having gone extinct is 50 and 95%, respectively, is shown in Fig. 2 (B and C). The median time for stem-group extinction increases as the time of crown-group origin increases, which is also true of the upper 95% probability, although this reaches a plateau before the upper end of our crown-group age distribution. The widths of the ranges between which the stem-group has a 95% chance of going extinct (Fig. 2D) are also shown. Here, the range increases rapidly before plateauing when crown-groups emerge at 440 Ma. Evidently, the temporal extent to which stem- and crown-group representatives can co-occur varies considerably.

### Total-group ages under a fixed crown-group origin show stochastic variance

We asked what range of total-group ages can be generated under a fixed crown-group age with constant rates of speciation and extinction; this experiment explores whether the crown-group can be expected to approximate the age of the total-group. No trees are simulated in this approach. Instead, coalescence events were generated from the root of the crown-group backward, with waiting times drawn from a probability distribution based on the speciation rate. Each lineage that coalesced with our clade was then allowed to either survive or go extinct. If it survived to the present, the coalescence time of this lineage with the crown-group being investigated represented the total-group age corresponding to the crown-group being investigated.

**Fig. 3:**
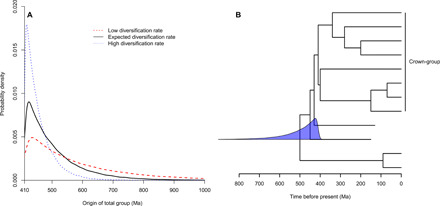
Ages of total-groups under a fixed crown-group age. (**A**) Probability densities of the total-group ages with different diversification rates. In all cases, extinction rate was set at 0.5 per species/million years. For low diversification rate, speciation rate was 0.5053. When the diversification rate was as expected, the speciation rate was 0.5107. When the diversification rate was high, speciation rate was 0.5214. (**B**) The distribution of total-group ages under the expected diversification rate shown on a hypothetical tree, with total-group originating at 500 Ma and crown-group originating at 410 Ma. This tree was not simulated and is present only to illustrate results.

First, we obtained a distribution of total-group ages under a fixed crown-group age of 410 Ma, an extinction rate of 0.5 per species/million years, and a speciation rate optimized based on a total-group age of 500 Ma (0.5107 per species/million years). The mean and median total-group ages were 503.3 and 474.7 Ma (*n* = 100,000) (Fig. 3), with 95% of total-groups with ages that fall within the range of 754.1 to 412.4 Ma.

As long as the diversification rate (speciation rate – extinction rate) remained the same, the effect of changing speciation rate was negligible. Halving the speciation rate to 0.2553 placed total-groups between 752.4 and 412.4 Ma; doubling it to 1.0214 resulted in total-group ages ranging from 754.1 to 412.3 Ma (*n* = 100,000 in both cases).

Changing the diversification rate had a greater effect. When the diversification rate was halved, by fixing speciation rate to 0.5053 speciation events per species/million years, the average length of the stem increased so that 95% of total-groups originated within the interval 1073.5 to 414.2 Ma (Fig. 2). By doubling the diversification rate (speciation rate = 0.5214), total-group ages ranged from 583.6 to 411.2 Ma (Fig. 3).

### Estimates for the age of the arthropod total-group vary stochastically under birth-death models

We explored the range of total-group ages that can be generated on the basis of constraints from phylum Arthropoda (Chelicerata + Mandibulata), the early fossil record of which is perceived to be among the best preserved, characterized, and understood of all the animal phyla (*9*). These simulations provide empirical context to our, so far, entirely hypothetical simulations, facilitating comparison to molecular clock estimates and interpretations of the arthropod fossil record. Initially, we constrained the age of the crown-group to 514 Ma [the minimum constraint on the age of the arthropod crown-clade (*10*)] and generated coalescent lineages back in time, as above. The extinction rate was set to 0.5 per species/million years. To define the speciation rate, we used a 535 million years estimate of total-group age (*9*) and an expectation of 7 million tips (*11*). These parameters, alongside the extinction rate, were used to calculate the maximum likelihood value of the speciation rate, which was 0.5237 events per species/million years. The 95% distribution of simulated total-group ages from a birth-death process (BDP) with these parameters span 670.3 to 515.1 Ma (mean, 556.3 Ma; median, 543.4 Ma; *n* = 100,000).

**Fig. 4:**
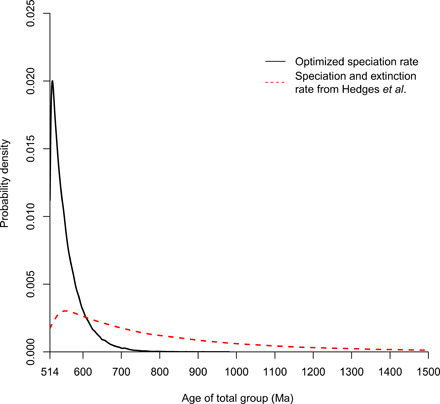
Arthropod total-group ages under a fixed crown-group age. Probability density of simulated total-group ages when the crown-group was fixed at 514 Ma is shown. The optimized speciation rate was 0.5237, and extinction rate was 0.5 events per species/million years. The speciation and extinction rates used from Hedges *et al.* (*12*) were 0.073 and 0.070 events per species/million years, respectively.

To explore the robustness of these results, the parameters of the BDP were changed. First, the speciation rate was set so that the expected number of tips was 14 million over 535 million years (0.5251 speciations per species/million years). Second, we explored the effect of changing the speciation rate to correspond to total-group ages between 1000 and 514 Ma. The speciation rate was 0.5120 and 0.5248, respectively, in these cases. When we conditioned on 14 million extant species, the total-group age was less than 633.3 Ma in 95% of cases. When the speciation rate was conditioned on an expected age of 1000 Ma, the upper 95 percentile total-group age was 764.1 Ma. When speciation rate was estimated to generate the clade at 514 Ma, the upper 95% bound on total-group age was 636.0 Ma. These results very closely resemble the probability distribution for the first set of simulations (Fig. 4, black solid line).

Last, we explored the impact of nonarbitrary diversification rates by fixing the speciation and extinction rates based on the constant rate birth-death model fitted to the eukaryote timetree by Hedges *et al.* (*12*). As our work and that of Budd and Mann (*8*) are predicated on constant rates of speciation and extinction, these values are potentially more appropriate than those estimated from analysis of arthropod clades [see (*13*)], which are likely to be higher than the underlying diversification rate, if such a rate exists. Here, the speciation rate was 0.073 speciation events per species/million years; extinction rate was 0.070 per species/million years. On the basis of these values, the mean, median, and upper 95% bound age for the arthropod total-group are 809.9, 708.1, and 1438.2 Ma.

## DISCUSSION

In the analyses presented above, we use a mixture of simulations and mathematically derived probability distributions to effectively relax some of the variables fixed by Budd and Mann (*8*) and show that their conclusions—that crown-groups form shortly after total-groups and that stem-groups go extinct shortly after the emergence of the crown-group—are conditional on the limiting assumptions implemented in their analyses. We arrive at different conclusions because our implementation enables us to explore the variance in results caused by stochastic effects. Our results show that there is a large range of diversification timings that can be generated under fixed rates of speciation and extinction. This broad range encompasses not only the young ages inferred in (*8*) but also much older ages. Hence, the implications that Budd and Mann (*8*) derived for interpreting the fossil record of major radiations and the veracity of molecular clock methodologies can be rejected.

### Clade antiquity cannot be predicted from the co-occurrence of stem- and crown-groups

In the first instance, Budd and Mann (*8*) concluded that, for diverse clades (≥10,000 extant species), crown clades emerge early in the evolutionary history of their total-group. In their example, the crown clade emerged 90 Ma after the establishment of the 500 Ma total-group. However, their analyses were conditioned on both the age of the crown- and total-group and, hence, could not inform the timing of emergence of crown clades relative to total-group clades. Our initial simulations were, conditioned only on the age of the total-group at 500 Ma, allowing us to estimate the relative timing of origin of crown clades. This demonstrated that, while crown clades of diverse lineages generally emerge soon after their ancestral total-group, our simulations reveal a broad distribution of crown clade ages (95% highest posterior density (HPD): 3.3 to 142.9 million years relative to 500 Ma total-group and 10 K extant species). Furthermore, variance is inversely proportional to extant clade diversity and diversification rate. We also show that, for diverse extant clades (≥10 K extant species), extending these simulations by a further 500 million years has little impact on the absolute difference between the timing of the emergence of the total-group and the crown-group. This naturally reflects the diversity-dependent extinction probability of the lineages that define the crown-group—the more diverse the clade, the lower the chance it will go extinct by stochastic extinction of species.

Second, we show that, while presumptive stem-groups often go extinct soon after the emergence of their crown-group, there is a broad variance in this relationship (there is a 95% chance the stem-group goes extinct within an interval 8 to 319 million years after the emergence of the crown-group at 410 Ma). A full description of this range, caused by the stochastic nature of the BDP, was omitted in (*8*), where results were reported only in terms of mean values across 50,000 replicate analyses. There is a correlation between the timing of origin of crown-groups and the subsequent survival of their presumptive stem-groups such that, for crown clades that emerge early in the evolutionary history of their total-group, the stem-group becomes extinct proportionally early and vice versa. Again, this is a diversity and time-dependent expectation.

In our third set of analyses, we showed that, when conditioning on the age of the crown clade, age estimates for the total-group also exhibit a broad distribution, extending well beyond the age on which the diversification rate was conditioned. In general, total-group ages approximate an exponential distribution with a lower limit equal to the age of the crown-group with shape dependent on the diversification rate. The higher the diversification rate, the shorter the time interval between crown-group and total-group origins. The reason for this is twofold. First, according to the speciation rate, waiting times between coalescence events should be shorter. Second, under a constant, nonzero extinction rate, a higher diversification rate means that it is more likely that a coalescent lineage will survive to the present.

For the most part, the diversification rates specified in our analyses do not reflect empirical estimates but match those used by Budd and Mann (*8*) to derive conclusions about the dynamics of stem- and crown-groups. With the exception of the speciation and extinction rates fitted to the eukaryote timetree (*12*), we use an extinction rate of 0.5 and speciation rates higher than this but always less than 0.6. It is difficult to estimate diversification rates in macroevolution due to the requirement of an accurate timetree, whether the timetree is estimated using fossil occurance, molecular data, or a combination of both. However, these figures are within the range of empirical estimates of other studies (*14*–*17*). We predict that, under lower diversification rates, the expected age of both total- and crown-groups would be older. Similarly, younger ages would be expected under higher diversification rates with all other parameters remaining the same.

Our analyses designed to reflect the diversity and fossil record of arthropods, perhaps the best preserved and characterized of evolutionary lineages, yield total-group ages that extend far back enough to encompass those estimated using molecular clock methods. Here, the age of the crown-group was fixed to the age of the oldest undisputed crown-arthropod [514 Ma; (*10*, *18*, *19*)], a conservative estimate of the age of crown-arthropods. Initially, the speciation rate was fixed such that the expected extant diversity of 7,000,000 species (*11*) achieved over 535 million years—a span of time chosen to reflect a conservative paleontological estimate of duration of the arthropod total-group (*20*). We show that total-group ages are exponentially distributed with the upper 95th percentile at 641.1 Ma; this result is robust to changes in the clade age used to estimate the speciation rate. We also used an empirical estimate of speciation and extinction rate based on a eukaryote timetree (*12*). The speciation, extinction, and diversification rates used here were much lower than in other analyses, and the range of total-group ages reflect this, extending much further into the past. Overall, using even the most conservative estimate of arthropod crown- and total-group clade ages shows that the null expectation of total-group age extends to before the Ediacaran. Most Bayesian molecular clock studies suggest an age of arthropods that is entirely consistent with this null expectation (*21*–*24*).

### Implications for interpreting the fossil record and the veracity of molecular time scales

When estimating divergence times, the molecular clock is applied, usually using genomic sequence data alongside information from the fossil record to infer a probability distribution of the age of clades throughout the tree being investigated. Where we have fixed either crown or total-group age, in reality, we do not know when total-groups emerge, which is the crux of the debate surrounding issues like the Cambrian explosion hypothesis (*25*, *26*). Instead, we can constrain the minimum possible age of clades based on their oldest fossil records (*27*) and a “soft” (i.e., nonlimiting but beyond which there is an extremely low probability of clade existence) maximum constraint based on an absence of fossil evidence despite the required conditions for fossilization being satisfied. If the molecular evidence is strong, then soft maxima can be violated. Thus, our analyses inferring total-group ages are analogous to recent molecular clock analyses of arthropod and animal evolution.

Budd and Mann (*8*, *20*) argued that, since their analyses implied that crown-groups emerge soon after total-groups, and stem-groups become extinct soon thereafter, it is possible to diagnose the progress of diversification dynamics from the stratigraphic occurrence of stem- and crown-group fossil representatives, at least for diverse extant clades (≥10 K species). In particular, if stem- and crown-group fossils co-occur stratigraphically, then the crown- and total-groups cannot be much older (*8*, *20*). However, as we have shown, these views are artifacts of experimental design and interpretation of their results. A key consequence of our observations on the stochastic nature of crown-group origin and stem-group longevity is that it is not possible to diagnose the progress of total-, stem-, and crown-group diversification based on the fossil record alone.

In conclusion, following Budd and Mann (*8*, *20*, *28*), we present expectations under a null model—that is, one of constant speciation and extinction across the entire tree. We do not investigate the effect of mass extinctions or varying rates of speciation and extinction which may better explain the timetree of life (*12*). If molecular clock estimates did exceed the expectations of this null model, an alternative model would have to be invoked to explain the findings [e.g., (*29*, *30*)]. However, the results of our simulations indicate that this is not currently necessary and that the molecular evidence and null expectations of tree shape are not in disagreement. Hence, the source of the mismatch between estimates of timings of major radiations—including modern mammals, birds, flowering plants, and bilaterians—based exclusively on the fossil record, and those estimated from a combination of molecular and fossil data must lie elsewhere, either with the fossil record or how we model sequence evolution. Evolutionary biologists can choose to read the fossil record more or less literally (*20*). However, we argue that it requires interpretation, and molecular clock methodologies provide means of achieving this by combining the best qualities of molecular and paleontological data that, after all, represent parallel and complementary records of the same evolutionary history. 

## MATERIALS AND METHODS

### Mathematical symbols

λ denotes the speciation rate, μ denotes the extinction rate, *t* denotes the time, *n*_*t*, *t*^′^_ denotes the number of species at time *t*^′^ in in a BDP beginning time *t*, and *s*_*t*, *t*^′^_ denotes the probability of a lineage at time *t* surviving to time *t*^′^.

#### Crown-group ages

The function sim.bd.taxa.age from the TreeSim R package (*31*) was used with fixed speciation rate, extinction rate, time since the BDP starts, and number of tips. The speciation rate was chosen as the value whose expected number of tips was equal to the desired number of tips to be simulated. The expected number of tips provided speciation rate > extinction rate is given in (Eq. 1) (*8*)

$$E(n_{0,t})=\lambda \frac{1-\text{exp}(-t(\lambda -\mu )\;)}{\lambda -\mu \text{exp}(t(\lambda -\mu ))}$$( 1 )

For the first analysis, λ = 0.5107, μ = 0.5, *n*_0,500_ = 10,000. For investigating smaller crown clades, λ = 0.505, μ = 0.5, *n*_0,500_ = 1000; and for testing the origin of larger crown clades, λ = 0.516, μ = 0.5, *n*_0,500_ = 100,000.

#### Stem-group extinction

Crown and stem-group lineages were enumerated using the method of Budd and Mann (*8*) . We modified their *R* script so that it ran only one analysis at a time, instead of the 50,000 which were simulated originally. We return a Boolean vector from 0 to 500, representing points in time, equal to “1” if the stem-group had at least one lineage and “0” if it did not. After 50,000 runs at each crown-group age, results were converted to a probability vector, representing the likelihood that the stem had 0 lineages (was extinct). This was done by dividing the number of simulations where the stem was extinct by the total number of simulations for each time point. For full details of the mathematical methods, see (*8*). For all analyses estimating stem group extinction, λ = 0.5107, μ = 0.5, *n*_0,500_ = 10,000.

#### Total-group ages

To find the distribution of total-group ages under a fixed crown-group age, coalescence events were generated back in time under a birth model. Waiting times were randomly drawn from an exponential distribution with rate equal to the speciation rate. When a coalescence event occurred, it indicated the origin of the total-group with probability equal to the probability that it survived to the present, given by Eq. 2

$$s_{t,t\prime }=\frac{\lambda -\mu }{\lambda -\mu \text{exp}(-(\lambda -\mu )(t\prime -t))}$$( 2 )

where *t* is the time of the coalescence event and *t*^′^ is the present. Coalescence events continued to be generated backward in time under the model until one survived to the present by drawing a random number between 0 and 1 if it less than or equal to the survival probability. According to the approach used here, the diversity of the crown group is irrelevant—only the speciation rate, extinction rate, and time of crown-group origin matter, so simulation of trees is not necessary. Thus, we do not simulate any trees when simulating total group ages.

For analyses with results summarized in Fig. 3A, for low diversification rate, λ = 0.5053, μ = 0.5; for the expected diversification rate, given the number of species, λ = 0.5107, μ = 0.5; and for the high diversification rate, λ = 0.5214, μ = 0.5

For analyses presented in Fig. 4 (plotted in black solid line), λ = 0.5237, μ = 0.5. For results plotted with a red dashed line, λ = 0.073, μ = 0.070. Graphics were generated using *R* (*32*).

## SUPPLEMENTARY MATERIALS

Supplementary material for this article is available at http://advances.sciencemag.org/cgi/content/full/7/24/eabf2257/DC1

## References

[1] G. G. Simpson, *Tempo and Mode in Evolution* (1944), pp. 237.

[2] J. L. Thorne, H. Kishino, I. S. Painter, Estimating the rate of evolution of the rate of molecular evolution. Mol. Biol. Evol. 15, 1647–1657 (1998). 9866200 10.1093/oxfordjournals.molbev.a025892

[3] A. J. Drummond, S. Y. Ho, M. J. Phillips, A. Rambaut, Relaxed phylogenetics and dating with confidence. PLOS Biol. 4, e88 (2006). 16683862 10.1371/journal.pbio.0040088 PMC1395354

[4] Z. Yang, B. Rannala, Bayesian estimation of species divergence times under a molecular clock using multiple fossil calibrations with soft bounds. Mol. Biol. Evol. 23, 212–226 (2005). 16177230 10.1093/molbev/msj024

[5] Z. Yang, Maximum likelihood phylogenetic estimation from DNA sequences with variable rates over sites: Approximate methods. J. Mol. Evol. 39, 306–314 (1994). 7932792 10.1007/BF00160154

[6] A. Cooper, R. Fortey, Evolutionary explosions and the phylogenetic fuse. Trends Ecol. Evol. 13, 151–156 (1998). 21238236 10.1016/s0169-5347(97)01277-9

[7] J. A. Cunningham, A. G. Liu, S. Bengtson, P. C. J. Donoghue, The origin of animals: Can molecular clocks and the fossil record be reconciled? Bioessays 39, 1–12 (2017). 10.1002/bies.201600120 27918074

[8] G. E. Budd, R. P. Mann, The dynamics of stem and crown groups. Sci. Adv. 6, eaaz1626 (2020). 32128421 10.1126/sciadv.aaz1626 PMC7030935

[9] A. C. Daley, J. B. Antcliffe, H. B. Drage, S. Pates, Early fossil record of Euarthropoda and the Cambrian Explosion. Proc. Natl. Acad. Sci. 115, 5323–5331 (2018). 29784780 10.1073/pnas.1719962115 PMC6003487

[10] J. M. Wolfe, A. C. Daley, D. A. Legg, G. D. Edgecombe, Fossil calibrations for the arthropod Tree of Life. Earth Sci. Rev. 160, 43–110 (2016).

[11] N. E. Stork, How many species of insects and other terrestrial arthropods are there on Earth? Annu. Rev. Entomol. 63, 31–45 (2018). 28938083 10.1146/annurev-ento-020117-043348

[12] S. B. Hedges, J. Marin, M. Suleski, M. Paymer, S. Kumar, Tree of life reveals clock-like speciation and diversification. Mol. Biol. Evol. 32, 835–845 (2015). 25739733 10.1093/molbev/msv037 PMC4379413

[13] J. Lozano-Fernandez, A. R. Tanner, M. N. Puttick, J. Vinther, G. D. Edgecombe, D. Pisani, A Cambrian–Ordovician terrestrialization of Arachnids. Front. Genet. 11, (2020). 10.3389/fgene.2020.00182 PMC7078165 32218802

[14] S. Magallón, M. J. Sanderson, Absolute diversification rates in angiosperm clades. Evolution 55, 1762–1780 (2001). 11681732 10.1111/j.0014-3820.2001.tb00826.x

[15] M. A. McPeek, J. M. Brown, Clade age and not diversification rate explains species richness among animal taxa. Amer. Natural. 169, E97–E106 (2007). 10.1086/512135 17427118

[16] J. P. Scholl, J. J. Wiens, Diversification rates and species richness across the Tree of Life. Proc. Biol. Sci. R. Soc. 283, 1838 (2016). 10.1098/rspb.2016.1334 PMC5031659 27605507

[17] L. F. Henao Diaz, L. J. Harmon, M. T. C. Sugawara, E. T. Miller, M. W. Pennell, Macroevolutionary diversification rates show time dependency. Proc. Natl. Acad. Sci. U.S.A. 116, 7403–7408 (2019). 30910958 10.1073/pnas.1818058116 PMC6462100

[18] X.-g. Zhang, D. J. Siveter, D. Waloszek, A. Maas, An epipodite-bearing crown-group crustacean from the Lower Cambrian. Nature 449, 595–598 (2007). 17914395 10.1038/nature06138

[19] R. C. M. Warnock, Z. H. Yang, P. C. J. Donoghue, Exploring uncertainty in the calibration of the molecular clock. Biol. Lett. 8, 156–159 (2012). 21865245 10.1098/rsbl.2011.0710 PMC3259980

[20] G. E. Budd, R. P. Mann, Survival and selection biases in early animal evolution and a source of systematic overestimation in molecular clocks. Interface Focus 10, 20190110 (2020). 32637066 10.1098/rsfs.2019.0110 PMC7333906

[21] M. S. Y. Lee, J. Soubrier, G. D. Edgecombe, Rates of phenotypic and genomic evolution during the Cambrian explosion. Curr. Biol. 23, 1889–1895 (2013). 24035543 10.1016/j.cub.2013.07.055

[22] O. Rota-Stabelli, A. C. Daley, D. Pisani, Molecular timetrees reveal a Cambrian colonization of land and a new scenario for ecdysozoan evolution. Current biology : CB 23, 392–398 (2013). 23375891 10.1016/j.cub.2013.01.026

[23] M. dos Reis, Y. Thawornwattana, K. Angelis, M. J. Telford, P. C. Donoghue, Z. Yang, Uncertainty in the timing of origin of animals and the limits of precision in molecular timescales. Curr. Biol. 25, 2939–2950 (2015). 26603774 10.1016/j.cub.2015.09.066 PMC4651906

[24] J. Lozano-Fernandez, R. Carton, A. R. Tanner, M. N. Puttick, M. Blaxter, J. Vinther, J. Olesen, G. Giribet, G. D. Edgecombe, D. Pisani, A molecular palaeobiological exploration of arthropod terrestrialization. Philos. Trans. R. Soc. Lond. B Biol. Sci. 371, 20150133 (2016). 27325830 10.1098/rstb.2015.0133 PMC4920334

[25] D. H. Erwin, M. Laflamme, S. M. Tweedt, E. A. Sperling, D. Pisani, K. J. Peterson, The Cambrian conundrum: early divergence and later ecological success in the early history of animals. Science 334, 1091–1097 (2011). 22116879 10.1126/science.1206375

[26] D. E. G. Briggs, The Cambrian explosion. Curr. Biol. 25, R864–R868 (2015). 26439348 10.1016/j.cub.2015.04.047

[27] J. F. Parham, P. C. J. Donoghue, C. J. Bell, T. D. Calway, J. J. Head, P. A. Holroyd, J. G. Inoue, R. B. Irmis, W. G. Joyce, D. T. Ksepka, J. S. L. Patané, N. D. Smith, J. E. Tarver, M. van Tuinen, Z. Yang, K. D. Angielczyk, J. M. Greenwood, C. A. Hipsley, L. Jacobs, P. J. Makovicky, J. Müller, K. T. Smith, J. M. Theodor, R. C. M. Warnock, M. J. Benton, Best practices for justifying fossil calibrations. Syst. Biol. 61, 346–359 (2012). 22105867 10.1093/sysbio/syr107 PMC3280042

[28] G. E. Budd, R. P. Mann, History is written by the victors: The effect of the push of the past on the fossil record. Evolution 72, 2276–2291 (2018). 30257040 10.1111/evo.13593 PMC6282550

[29] D. Jablonski, Lessons from the past: Evolutionary impacts of mass extinctions. Proc. Natl. Acad. Sci. U.S.A. 98, 5393–5398 (2001). 11344284 10.1073/pnas.101092598 PMC33224

[30] B. D. Barnes, J. A. Sclafani, A. Zaffos, Dead clades walking are a pervasive macroevolutionary pattern. Proc. Natl. Acad. Sci. U.S.A. 118, e2019208118 (2021). 33827921 10.1073/pnas.2019208118 PMC8053996

[31] T. Stadler, Simulating trees with a fixed number of extant species. Syst. Biol. 60, 676–684 (2011). 21482552 10.1093/sysbio/syr029

[32] R Core Team, *A Language and Environment for Statistical Computing* (R Foundation for Statistical Computing, Vienna, Austria, 2019).

